# The Challenge of Classifying Metastatic Cell Properties by Molecular Profiling Exemplified with Cutaneous Melanoma Cells and Their Cerebral Metastasis from Patient Derived Mouse Xenografts*[Fn FN2]

**DOI:** 10.1074/mcp.RA119.001886

**Published:** 2019-12-31

**Authors:** Benjamin Neuditschko, Lukas Janker, Laura Niederstaetter, Julia Brunmair, Katharina Krivanek, Sivan Izraely, Orit Sagi-Assif, Tsipi Meshel, Bernhard K. Keppler, Giorgia Del Favero, Isaac P. Witz, Christopher Gerner

**Affiliations:** ‡ Department of Analytical Chemistry, Faculty of Chemistry, University of Vienna; § Department of Inorganic Chemistry, Faculty of Chemistry, University of Vienna; ¶ Department of Cell Research and Immunology, The George S. Wise Faculty of Life Sciences, Tel Aviv University; ‖ Department of Food Chemistry and Toxicology, Faculty of Chemistry, University of Vienna; ** Joint Metabolome Facility, Faculty of Chemistry, University of Vienna; ‡‡ Core Facility Multimodal Imaging, Faculty of Chemistry, University of Vienna

**Keywords:** Melanoma, metastasis, tumor microenvironment, mouse models, omics, brain metastasis, eicosanoids, xenograft model

## Abstract

The prediction of metastatic properties from molecular analyses still poses a major challenge. Here we aimed at the classification of metastasis-related cell properties by proteome profiling making use of cutaneous and brain-metastasizing variants from single melanomas sharing the same genetic ancestry. Previous experiments demonstrated that cultured cells derived from these xenografted variants maintain a stable phenotype associated with a differential metastatic behavior: The brain metastasizing variants produce more spontaneous micro-metastases than the corresponding cutaneous variants. Four corresponding pairs of cutaneous and metastatic cells were obtained from four individual patients, resulting in eight cell-lines presently investigated. Label free proteome profiling revealed significant differences between corresponding pairs of cutaneous and cerebellar metastases from the same patient. Indeed, each brain metastasizing variant expressed several apparently metastasis-associated proteomic alterations as compared with the corresponding cutaneous variant. Among the differentially expressed proteins we identified cell adhesion molecules, immune regulators, epithelial to mesenchymal transition markers, stem cell markers, redox regulators and cytokines. Similar results were observed regarding eicosanoids, considered relevant for metastasis, such as PGE2 and 12-HETE. Multiparametric morphological analysis of cells also revealed no characteristic alterations associated with the cutaneous and brain metastasis variants. However, no correct classification regarding metastatic potential was yet possible with the present data. We thus concluded that molecular profiling is able to classify cells according to known functional categories but is not yet able to predict relevant cell properties emerging from networks consisting of many interconnected molecules. The presently observed broad diversity of molecular patterns, irrespective of restricting to one tumor type and two main classes of metastasis, highlights the important need to develop meta-analysis strategies to predict cell properties from molecular profiling data. Such base knowledge will greatly support future individualized precision medicine approaches.

## Abstract (precis)

Proteome profiling data of eight xenografted cutaneous and cerebellar metastasis of human melanoma cells were combined with eicosanoid and glutathione measurements as well as multiparametric morphologic analyses and immunofluorescence. The analyses revealed significant differences in the expression of molecules associated with metastatic properties, while lacking any commonalties when comparing the metastatic variants from four different donors. Apparently we lack appropriate meta-analysis strategies making use of the large number of presently identified metastasis-associated molecules for successful classification of cells.

## Graphical Abstract

**Figure:**
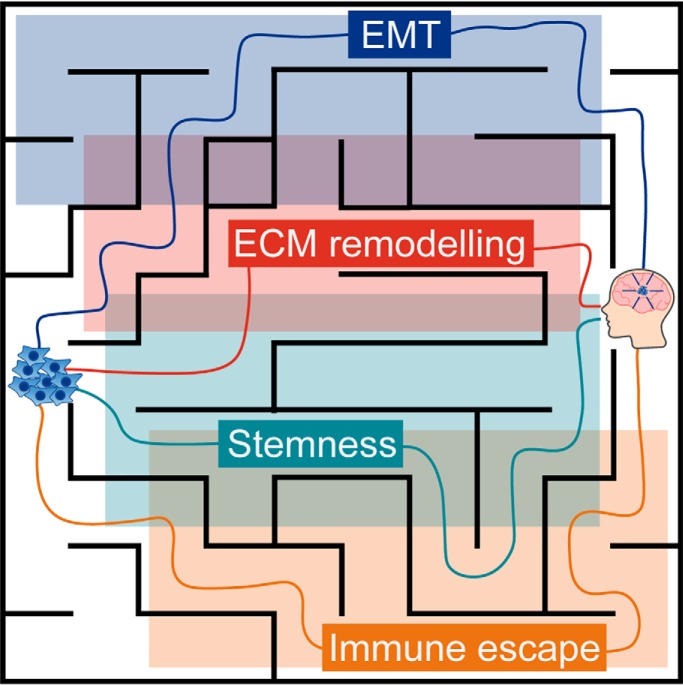

- Comprehensive molecular profiling of cutaneous and cerebellar metastasis variants.
- Identification of differentially regulated metastasis-associated molecules.
- Evidence for individually distinct patterns of metastasis-associated molecules.
- Highlighting the evident need for establishing meta-analyses strategies.

Molecular profiling of cells aims at the prediction of relevant cell properties eventually supporting the optimal choice of therapy based on individualized data related to a given patient. In fact, the prediction of cell properties directly related to the expression of a defined set of functional proteins such as *e.g.* matrix metalloproteinases mediating the degradation of extracellular matrix, already works in a reliable fashion (1). However, the prediction of complex cell properties rather indirectly related to an unknown number of mediators, such as malignant phenotypes of metastatic diseases, still poses major challenges. In this work we made use of metastatic melanoma cells with stable and well-described patterns of metastasis to evaluate whether and how such prediction of cell properties out of molecular profiling data might become feasible.

The incidence of brain metastasis in melanoma patients is one of the highest for all tumors and it is generally associated with poor prognosis (2). Moreover, brain metastasis generally relates to intrinsic drug resistance properties (3). Investigating genetic traits of such tumor cells revealed a significant genetic diversity among melanoma tumors associated with high mutation rates, eventually accounting for the present difficulties in understanding the underlying mechanisms of metastasis (4). To draw general statements on the molecular events sustaining the development of metastasis proves to be a very challenging task, sometimes associated with apparently contradicting conclusions. Remarkably, genetic traits of melanoma cells also hardly correlate with survival or with the time from primary diagnosis to the detection of brain metastasis (5). Thus, the absence or presence of certain mutations in key molecules such as BRAF, NRAS or KIT is not directly related to the capability of melanoma cells to colonize the brain (5). This was our motivation to apply post-genomic techniques searching for molecular patterns potentially associated with brain metastasis which might also support the functional understanding of accompanying drug resistance properties. We have previously applied proteome profiling to investigate melanoma drug resistance features as well as melanoma brain metastases (6–8).

To systematically investigate potential molecular patterns associated with brain metastasis, we have used stable and well-described melanoma cell models originating from four different patients. Primary melanoma cells isolated from the patients were xenografted into nude mice and repeatedly inoculated into either the hypoderm or the brain thus establishing human melanoma xenograft models encompassing four pairs of local (cutaneous - C variants) and brain metastasis variants (CB variants) (9). Stable phenotypes were obtained and subsequently characterized (10–12). Each corresponding pair of cutaneous and brain metastasis variants share an identical genetic background. Any molecular difference between C and CB cells of each pair may thus be mainly attributed to post-genomic differences between these variants originating from cellular adaptation to different microenvironments. Certain CB variants spontaneously migrate into the brain when inoculated subdermally (9) suggesting that these variants may have gained stable brain metastasizing properties.

The subsequent molecular profiling analysis of these variants was designed to support two independent strategies. First, the large number of identified proteins allowed us to specifically investigate how known molecular players are expressed in these models. Cell functions known to be related to metastasis comprising migration, intravasation, survival in circulation and extravasation through the blood brain barrier (13) were considered with priority. Second, statistical analysis was performed to search for potentially unknown molecules significantly associated with metastatic properties. The results demonstrate that indeed the applied molecular profiling methods revealed many apparently meaningful molecular alterations associated with the metastatic variants, supporting a potential classification of cells according to metastasis-related molecules. However, no molecular pattern could be ascertained which would support an unequivocal classification regarding the known metastatic phenotypes of the cells. The data suggest that current classification strategies are not yet capable of predicting relevant cell properties in a satisfying fashion. We present evidence for the establishment of largely individual and specific strategies for metastasis on adaptation which need to be comprehended accordingly to make molecular profiling efficient for individualized precision medicine.

## MATERIALS AND METHODS

### 

#### 

##### Cell Culture Conditions and Sample Preparations

Cutaneous human melanoma cells (YDFR-C, DP-C, M12-C and M16-C) and brain metastatic human melanoma cells (YDFR-CB, DP-CB, M12-CB and M16-CB) were previously obtained as described previously (9). Briefly, human melanoma cells from four different melanoma patients were inoculated subdermally in nude mice to generate cutaneous melanoma tumors. Tumors were excised, minced into single cell suspension, and put in culture, until a pure melanoma culture was obtained, representing the cutaneous (C) variants. These cells were then inoculated intracardially 2–3 times into nude mice, each time resulting in the isolation and culturing of brain metastases, until a pure melanoma culture was obtained, representing the brain metastatic (CB) variants. These cells were subsequently cultured in RPMI 1640 (1X with l-Glutamine; Gibco, Thermo Fischer Scientific, Austria) with 10% (v/v) heat inactivated fetal calf serum (FCS^1^, Sigma-Aldrich, Austria) and 1% (v/v) Penicillin and Streptomycin (Sigma-Aldrich) in humidified incubators at 37 °C and 5% CO_2_. Cells were grown in T25 polystyrene cell culture flasks with cell growth surface for adherent cells (Sarstedt, Austria) and sub-cultured every 3–4 days. Therefor the medium was removed, the cells were washed twice with 5 ml PBS and incubated with 1.5 ml Trypsin-EDTA solution 0.25% (Sigma-Aldrich) for 3 min. The reaction was stopped by adding 4 ml fully supplemented medium and centrifuged for 5 min at 220 × *g*. After removing the supernatant, the cells were re-suspended in fully supplemented medium and seeded in a ratio of 1:6. Cells were regularly tested for absence of mycoplasma contamination and consistency in cell growth and morphology (*i.e.* size distribution and aggregation coefficient) was monitored during sub-culturing as well during the experiments with CASY TT Cell Counter and Analyzer (OMNI Life Science GmbH & Co. KG, Bremen, Germany). Cells were counted with a MOXI Z Mini Automated Cell Counter (ORFLO Technologies) using Moxi Z Type M Cassettes (ORFLO Technologies) and the number of seeded cells for the experiments calculated based of these results. For the performance of the assays, cells were used between passage 2 and 25.

##### Animals

Male athymic nude mice (BALB/c background) were purchased from Harlan Laboratories Limited (Israel). The mice were housed and maintained in laminar flow cabinets under specific pathogen-free conditions in the animal quarters of Tel Aviv University and in accordance with current regulations and standards of the Tel-Aviv University Institutional Animal Care and Use Committee. The mice were used when they were 7–8 weeks old.

##### Orthotopic Inoculation of Tumor Cells and Metastasis Formation Assays

1 × 10^6^ melanoma cells in 100 μl of 5% FCS supplemented RPMI 1640 medium were inoculated subdermally into the right thigh of male nude BALB/c mice. Brains were harvested 2 months following the inoculation and immediately frozen and stored at −70 °C, until used for RNA extraction. Detection of human cells (micrometastases) in mouse brain by RT-qPCR was performed as described previously (9).

##### Proteomics of Cytoplasmic and Nuclear Fractions

For the proteomics analysis of the cytoplasmic and nuclear fraction 2 × 10^6^ cells of passage 2 to 4 were seeded in a T25 cell culture flask with 3 ml of fully supplemented medium. After 24 h incubation the cells were lysed as previously described (14). In short, the medium was removed and the cells were carefully washed with 5 ml PBS twice. The lysis buffer was added onto the cells and lysed by pushing the suspension through a needle, centrifuged and the supernatant was precipitated in ice cold ethanol (EtOH, abs. 99%; AustroAlco, Austria). The remaining nuclear pellet was lysed with a Tris-EDTA, NP-40 buffer and after centrifugation the supernatant was again precipitated and kept on −20 °C over night.

##### Supernatant Analysis of Eicosanoids and Proteomics

2 × 10^6^ cells of passage 4 to 6 were seeded in a T25 cell culture flask with 3 ml fully supplemented medium and kept in the incubator. After 24 h the supernatant was removed, spiked with 10–100 nm of each internal standard (supplemental Table S1), centrifuged with 726 × *g* and 3 ml precipitated with 12 ml ethanol (EtOH, abs. 99%; AustroAlco) and stored at −20 °C over night. Precipitated proteins containing fetal calf serum proteins were discarded, while the eicosanoids were isolated from the supernatant. The cells were washed twice with 5 ml PBS and 3 ml of RPMI without supplements was added. After 4 h incubation the supernatant was removed, centrifuged and precipitated in ethanol overnight to obtain the secreted proteins.

##### Protein Sample Preparation

The proteins were used for a filter-assisted protein digest as described previously (15). In short, the precipitated proteins were centrifuged at 4536 × *g* for 30 min, the supernatant discarded and the protein pellet dried. After dissolving in sample buffer the protein concentration was determined and 20 μg of total protein was used for the digestion. After reduction with dithiothreitol and alkylation with iodacetamid (both Sigma-Aldrich) proteins were digested with Trypsin/Lys-C (MS grade; Promega Corporation, Madison, WI) and dried via vacuum centrifugation.

##### Eicosanoid Extraction

Samples were centrifuged (30 min, 4536 × *g*, 4 °C) and the supernatant transferred to a new 15 ml Falcon tube. Ethanol was evaporated via vacuum centrifugation at 37 °C until the original sample volume was restored. Samples were loaded on conditioned 30 mg/ml StrataX solid phase extraction (SPE) columns (Phenomenex, Torrance, CA). Columns were washed with 2 ml MS grade water and eicosanoids were eluted with 500 μl methanol (MeOH abs.; VWR International, Vienna, Austria) containing 2% formic acid (FA; Sigma-Aldrich). MeOH was evaporated using N_2_ stream at room temperature and reconstituted in 150 μl reconstitution buffer (H_2_O/ACN/MeOH + 0,2% FA − 65:31.5:3.5), containing a set of internal eicosanoid standards at a concentration of 10–100 nm (supplemental Table S1).

##### Analysis of Reduced and Oxidized Glutathione

5 × 10^5^ cells of passage 10–12 were seeded in a 6-well polystyrene plate with cell growth surface for adherent cells (Sarstedt) with 1 ml fully supplemented medium and allowed to grow for 24 h. Cells were washed twice with 1 ml PBS. After that, 500 μl of 2.5 mm N-Ethylmaleimide (NEM, Sigma-Aldrich) in PBS was added to each well. Samples were kept in the dark for 20 min at room temperature. The NEM-PBS solution was removed; cells were washed twice with 1 ml PBS. Two hundred microliters of 80% MeOH were pipetted to each well and incubated on ice for 20 min. Cells were scraped off, transferred into an Eppendorf tube and spun down for 10 min at 13,000 × *g* and 4 °C. The supernatant was moved to a 500 μl Eppendorf Tube, dried under nitrogen stream and then re-suspended in 100 μl 50 mm ammonium acetate (NH_4_OAc; pH = 7; Sigma-Aldrich). Three hundred microliters dichloromethane were added, and samples were vortexed. Next, samples were centrifuged for 5 min at 13,000 × *g* and 4 °C. Forty-five microliters of the upper phase were transferred to a new tube and 2.5 μl of 5 mm Tris(2-carboxyethyl)phosphine hydrochloride (TCEP; pH = 7; Sigma-Aldrich) was added. Samples were left on a thermoshaker for 30 min at 37 °C. Afterward, 2.5 μl of 100 mm N-Ethyl-D5-Maleimide (D5-NEM, Sigma-Aldrich) were added and samples were again placed in the dark for 20 min at room temperature. Finally, samples were diluted 1:5 in 50 mm NH_4_OAc and stored at 4 °C until further analysis.

##### HPLC-MS/MS for Proteomics

For the HPLC-MS/MS analysis the peptides were resolved in 5 μl 30% formic acid and diluted with 40 μl of mobile phase A (97.9% H_2_O, 2% acetonitrile, 0.1% formic acid). 1 μl for the supernatant samples and 10 μl of cytoplasmic and nuclear samples were injected into the Dionex UltiMate 3000 RSLCnano LC system coupled to the QExactive Orbitrap MS (all Thermo Fisher Scientific). Peptides were trapped on a C18 2 cm × 100 μm precolumn and LC separation was performed on a 50 cm × 75 μm Pepmap100 analytical column (both Thermo Fisher Scientific). For the supernatant samples 1 μl of sample was injected. The 85 min HPLC method included a 43 min gradient from 7% to 40% mobile phase B (79.9% acetonitrile, 20% H_2_O, 0.1% formic acid) at a flow rate of 300 nL/min. For the cytoplasmic and nuclear samples 10 μl were injected. The 135 min HPLC method included a 95 min gradient from 8% to 40% mobile phase B at a flow rate of 300 nL/min. Mass spectrometric settings were the same for all fractions. The resolution on the MS1 level was set to 70,000 (at *m*/*z* = 200) with a scan range from 400 to 1400 *m*/*z*. The top eight abundant peptide ions were chosen for fragmentation at 30% normalized collision energy and resulting fragments analyzed in the Orbitrap at a resolution of 17,500 (at *m*/*z* = 200).

##### Proteomics Data Analysis

Raw data were subjected to the freely available software MaxQuant (version 1.6.0.1) utilizing the Andromeda search engine, followed by statistical evaluation with the Perseus software (version 1.6.0.2). For the MaxQuant search, a minimum of two peptide identifications, at least one of them being a unique peptide, was required for valid protein identification. Digestion mode was set to “Specific” choosing Trypsin/P. The peptide mass tolerance was set to 50 ppm for the first search and to 25 ppm for the main search. The false discovery rate (FDR) was set to 0.01 both on peptide and protein level, based on the q-value. The database applied for the search was the human Uniprot database (version 03/2018, reviewed entries only) with 20,316 protein entries. Further settings for the search included carbamidomethylation as fixed modification and oxidation of methionine and acetylation of the protein C terminus as variable modifications. Each peptide was allowed to have a maximum of two missed cleavages and two modifications, “Match between runs” was enabled and the alignment window set to 10 min, with the match time window of 1 min.

##### UHPLC-MS/MS for Eicosanoid Measurements

Analytes were separated using a Thermo Scientific Vanquish (UHPLC) system and a Kinetex^®^ C18 - column (2.6 μm C18 100 Å, LC Column 150 × 2.1 mm; Phenomenex^®^). Applying a 20 min gradient flow method, starting at 35% B steadily increasing to 90% B (1–10min), going up to 99% B in 0.25 min. Flow rate was kept at 200 μl/min, 20 μl injection volume and column oven temperature was set to 40 °C. Eluent A contains H_2_O + 0.2% FA and eluent B ACN:MeOH (90:10) + 0.2% FA.

Mass spectrometric analysis was performed with a Q Exactive HF Quadrupole-Orbitrap mass spectrometer (Thermo Fisher Scientific), equipped with a HESI source for negative ionization. Mass spectra were recorded operating from 250 to 700 *m*/*z* at a resolution of 60 000 @ 200 m/z on MS1 level. The two most abundant precursor ions were selected for fragmentation (HCD 24 normalized collision energy), preferentially molecules from an inclusion list which contained 32 *m*/*z* values specific for eicosanoids (supplemental Table S2). MS2 was operated at a resolution of 15,000 @ 200 *m*/*z*. For negative ionization, a spray voltage of 2.2 kV and a capillary temperature of 253 °C were applied, with the sheath gas set to 46 and the auxiliary gas to 10 arbitrary units.

Raw files generated by the Q-ExactiveTM OrbitrapTM were analyzed manually using Thermo Xcalibur 4.1.31.9 (Qual browser), comparing reference spectra from the Lipid Maps depository library from July 2018 (3). For peak integration the software TraceFinderTM (version 4.1 - Thermo Scientific) was used.

##### UHPLC-MS/MS for Targeted Glutathione Measurements

Chromatographic separation was performed on a Kinetex 2.6 μm XB-C18 100Å (100 × 2.1 mm) column using a 1290 Infitiny UHPLC System (Agilent Technologies). Mobile phase A consisted of water with 0.2% formic acid and mobile phase B of methanol with 0.2% formic acid. The following gradient profile was run: 0 to 30% B in 5 min, 30 to 70% B in 2 min, afterward washed for 3 min at 95% B and at 10.1 min the system was reequilibrated at 0% B giving a total runtime of 12 min. Flow rate was 0.3 μl/min, the column temperature was set to 40 °C and the injection volume was 5 μl. MS detection was performed in the positive ion mode on a triple quadrupole mass spectrometer (QqQ 6490, Agilent technologies).

ESI spray voltage was 4500V, gas flow 12 L/min, gas temperature 250 °C, sheath gas temperature 400 °C, sheath gas flow 12 L/min, and nebulizer gas pressure 35 psi.

MRM transitions and the collision energies are summarized in supplemental Table S3. As quantifier in the final analysis of GSH-NEM the transition from *m*/*z* 433 to 84.2 was used, for GSH-d5NEM the transition from *m*/*z* 438 to 84.2 was used. The other transitions were used as qualifiers to ensure identity of the analytes.

GSH-NEM is a diastereomer, therefore GSH-NEM and GSH-d5NEM elute as two peaks because they have different physical and chemical properties (16). The areas under both peaks were summarized and used for integration and quantification. The obtained raw data was processed with Skyline (MacCoss Lab Software).

##### Experimental Design and Statistical Rationale

Three independent cell culture experiments were carried out for every cell line. All samples were analyzed with LC-MS/MS in 2 technical replicates which resulted in 144 measurements for the proteomics, 48 measurements for eicosanoids and 48 measurements for glutathione. The proteins were identified using MaxQuant software (version 1.6.0.1), including the Andromeda search engine. For statistical evaluation, the Perseus software (version 1.6.0.2) was used. Potential contaminants, reversed sequences and proteins identified only by site were removed. The normal distribution of the data was manually checked using the histogram function. After the Label-free quantification (LFQ) values were logarithmized to base 2, the technical replicates were averaged. Invalid values were filtered with the criteria that at least 70% of the samples per group, being each individual cell line, have valid values. After application of these procedures, a final list of 5665 protein identifications regarding cytoplasmic and nuclear fractions and 1238 proteins in the supernatants was obtained. The still missing LFQ values were then replaced from a normal distribution with a down shift of 1.8 and a width of 0.3 to enable t-tests. Volcano plots were created to analyze the significantly regulated proteins (FDR < 0.05, S0 = 0.1). Identified proteins as well as statistical values of the t-tests are available in supplemental Tables S5–S8. The heatmaps were generated by a custom R (https://www.r-project.org) script. OriginPro 2018b was used for the generation of the spiderweb plots (Fig. 4), for the bar plots (Fig. 3) as well as for the two-sided-t-tests for the eicosanoid data (S0 = 0.1) which is indicated in the heatmap (Fig. 2).

##### Morphological Evaluation Using Bright-field Microscopy

To obtain the multiparametric evaluation of the morphology of the melanoma variants, cells were seeded in 6-wells plates (polystyrene, Cell+ growth surface for adherent cell; Sarstedt AG & Co., Nümbrecht, DE). After 72 h settling time, bright field images were taken with an Olympus CKX53 Inverted Microscope (10× magnification). Images were analyzed manually deriving area, perimeter, minimum and maximal radius of the cells with the software CellSens Entry. Circularity (4π area/perimeter) and roundness (minimum radius/maximum radius) were calculated as previously described (17). For the purpose of the study, 8–10 cells were randomly chosen from 5–6 optical fields and the experiments were repeated in 5 independent cell preparations, resulting in the quantification of more than 200 cells for every condition. To avoid selection bias, image analysis was performed after acquiring all the images form all the experiments and quantification workflow was independently verified with the software Gen5 (BioTek, Winooski, VT). Determination of cell volume and diameter was performed as previously described with the cell counter analyzer CASY-TT (18). Briefly, cells were gently detached with trypsin and after inactivation of the enzyme 100 μl cell suspension was diluted in 10 ml CASYton solution. Measurement range threshold was optimized at the beginning of the experiments and kept constant for all the subsequent analysis. Measurements were performed in technical duplicates from 5 independent cell preparations. For the graphical representation of the results values were corrected through adjustment values as reported in supplemental Table S4 and the software Origin Pro 9.1G was used for the generation of the spiderwebs.

##### Immunolocalization Nrf2

For the localization of Nrf2, an immunofluorescence experiments were performed for all the melanoma variants as previously described (19, 20). Briefly, cells were seeded on μ-Slides (35000 cells/well, Ibitreat coating, ibidi GmbH Martinsried, Germany), fixed with pre-warmed formaldehyde (FA, 3.7%, 15 min) and permeabilized with Triton X (0.2%, 10 min). Blocking of unspecific binding sites was obtained with donkey serum (2%, 1h). For the localization of transcription factor a mouse monoclonal and Nrf2 antibody was used (ab 89443 Abcam, dilution 1:600, 2 h). Detection was obtained with a donkey anti mouse antibody (Alexa Fluor 488-conjugated, 715–545-150 Jackson ImmunoResearch, dilution 1:1000, 1.5 h). After washing, cells were post-fixed with FA (10 min.) and glycine (100 mm) was used to mask reactive sites. Slides were mounted and sealed with Roti-Mount FluoCare with DAPI to counterstain the nuclei (Roth, Graz, Austria). Images were acquired with a Confocal LSM Zeiss 710 equipped with ELYRA PS. 1 system. Structured illumination microscopy (SIM) images were acquired with a Plan Apochromat 63X/1.4 oil objective.

## RESULTS

### 

#### 

##### Unbiased Statistical Analysis of Proteome Profiles of Metastatic Variants

**Fig. 1.**
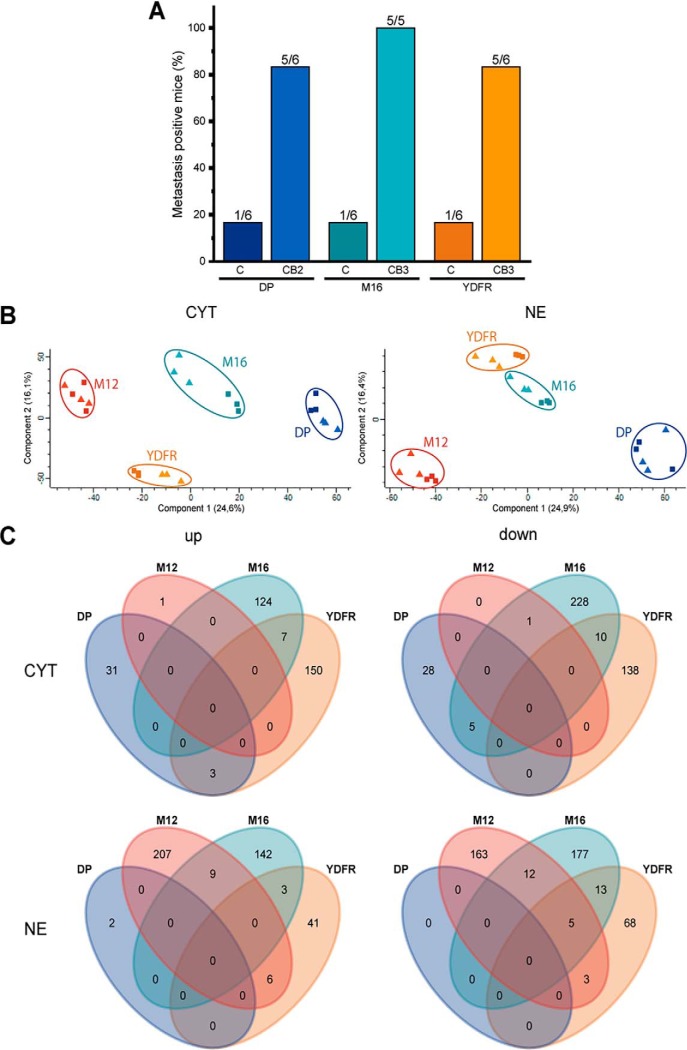
***A*, Percentage of mice with positive brain metastasis after subdermal inoculation with cultured melanoma cells as used for the present experiments.** *B*, Principal component analysis (PCA) for cytoplasmic (CYT) and nuclear fraction (NE). The C variants are represented by the rectangles whereas the CB variants are indicated by the triangles. *C*, Venn diagrams comparing all significant regulations indicated in the volcano plots (Fig. 2). The diagrams show the up- and down-regulated proteins from the cytoplasm and nuclear fraction separately.

Four pairs of melanoma cells representing cutaneous (C) and cerebellar (CB) variants of metastases, here in short referred to as DP, M12, M16 and YDFR, were analyzed for this study. The distinct metastatic potential of the CB and C variants, as used for the molecular profiling experiments, was successfully verified in case of three cell pairs as depicted in Fig. 1*A*. Almost all animals established brain metastases on subdermal inoculation of any of the CB variants, whereas only one out of six tested animals did so in case of the C variants. Label-free proteome profiling was performed with all four cell pairs after cell fractionation to obtain cytoplasm (CYT), nuclear extracts (NE) and cell supernatants (SN). The experimental workflow is depicted in supplemental Fig. S1.

A principal component analysis (PCA) of CYT and NE fractions revealed a clear distinction of all four cell pairs but rather incomplete separation of the C and CB variants (Fig. 1*B*). This would indicate that the variation between donors exceeds the variation observed between C and CB variants. Only in case of M16 and YDFR cells the variants were fully separated in both fractions, whereas the DP variants were only separated in the cytosolic fraction and the M12 variants only in the nuclear extracts. PCA of cell supernatants revealed similar results (supplemental Fig. S2*A*). Imaginary vectors in the PCA graphs pointing from the experiments representing C variants to experiments representing corresponding CB variants would point in different directions. These observations already indicated rather few commonalities shared by the CB variants representing a distinct metastatic phenotype.

**Fig. 2.**
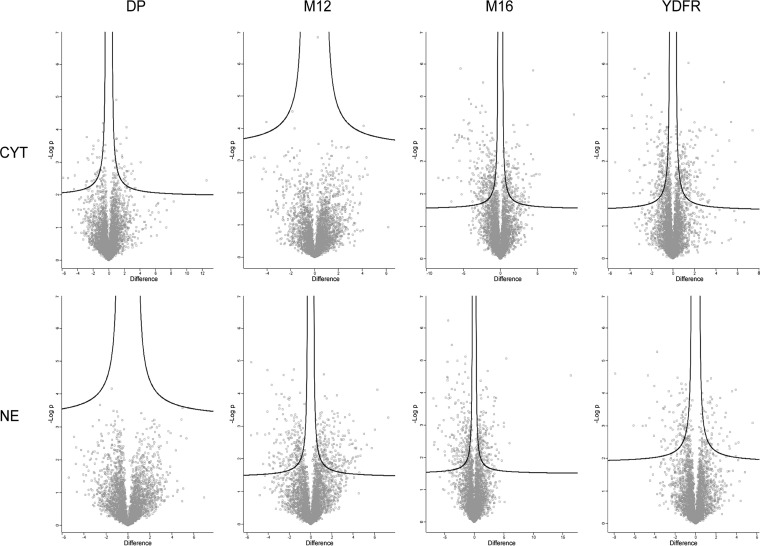
**Volcano plots for all 4 cell pairs comparing each C-CB pair (FDR < 0. 05, S0 = 0.1).** Up- or down-regulation corresponds to higher or lower LFQ intensities in the CB variant compared with the C variant, respectively.

Wondering whether we would be able to identify a proteome signature associated exclusively with the metastatic subgroup, a statistical analysis comparing all CB variants to all C variants was performed. However, not a single protein regulatory event was found apparently associated with a metastatic variant. Two scenarios might explain such a result. Either the experimental variation is too high rendering the power of the analysis insufficient or, alternatively, no specific commonality between the individual differences between C and CB variants would potentially be able to account for the distinct cell properties. Actually the experimental variation seemed to be rather acceptable, as all CB-C pairs displayed significant proteome alterations (FDR < 0.05) when compared with each other separately (Fig. 2). However, comparing the commonalities of these deregulated proteins between all pairs revealed only very little overlap (Fig. 1*C*). Although the CB variants of M16 and YFDR shared at least a small number of regulated proteins compared with the corresponding C variants, the other donors showed rather different regulatory events.

Although the strategies listed above were restricted to the consideration of significant regulatory events, we wondered whether some kind of co-regulated proteome signature would emerge considering all proteome alterations regardless of significance thresholds. To this aim, we blotted ranked difference levels between CB-C pairs and projected difference levels of the corresponding proteins derived from another CB-C pair. As illustrated in supplemental Fig. S3, no similarities between such regulatory patterns were becoming apparent. In addition, correlation analyses of all regulatory events regardless of significance levels between all potential pairs of donors was performed and depicted in supplemental Fig. S4. All R^2^ values of these correlation analyses were as little as ranging from 0.0001 to 0.05. These observations again supported the notion of a lack of commonalities of CB-related proteome alterations.

##### Specific Consideration of Metastasis-related Proteins Distinguishing CB and C Variants

To test the hypothesis that the CB variants may have altered the proteome profile in a biologically meaningful fashion providing them with relevant properties, we not only performed statistical analyses in an unbiased fashion as described above, but also focused on known metastasis-related proteins. In a first step, the question was investigated whether such proteins were found altered in any of the analyzed CB-C pairs. Thus, we started to investigate the significantly altered proteins depicted in Fig. 2 and listed in supplemental Tables S5–S8 whether they comprised such proteins.

**Fig. 3.**
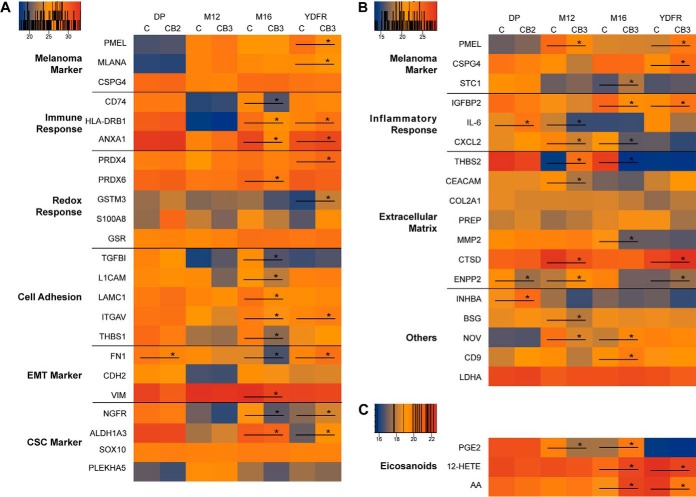
***A*, Heatmap for selected proteins of the cytoplasmic fraction for all cell lines.** *B*, Heatmap for selected proteins of the supernatant. Legend values are logarithmic LFQ intensities calculated by MaxQuant. *C*, Heatmap for the eicosanoids PGE2, 12-HETE and the precursor AA of the supernatant. Values correspond to logarithmic intensities normalized to the internal deuterated standards. Black bars with stars (*) indicate significant regulations (FDR < 0.05, S0 = 0.1) between C and CB variants.

Results of this analysis are visualized in heatmaps (Fig. 3). First, clinically relevant melanoma marker molecules PMEL and MLANA (21) were detected in all cells and found significantly down-regulated in the YDFR-CB variant. All detected melanoma markers varied substantially between the cells of the different donors, whereas CSPG4 (21, 22) was found uniformly increased in all four CB variants lacking significance in all of them. Proteins potentially regulating immune responses of the host such as class II histocompatibility antigens HLA-DRB1 and CD74 (23), and the innate immune response regulator ANXA1 were found specifically altered between the variants and between the donors with no consistent expression pattern. The same notion applies to cell adhesion molecules TGFBI, L1CAM, LAMC1, ITGAV, THBS1 (24), the EMT marker molecules FN1, CDH2 and VIM (25), and the cancer stem cell marker molecules NGFR (CD271), ALDH1A3, and PLEKHA5 (26) as depicted in Fig. 3*A*. In contrast, the cancer stem cell marker molecule SOX10 was rather uniformly expressed in all eight cells (Fig. 3*A*). The analysis of secretomes specifically identified inflammatory mediators such as interleukin-6 and the chemokine CXCL2, and a broad variety of extracellular matrix proteins (Fig. 3*B*). In addition, the lymphangiogenesis regulator INHBA (27), the fibroblast activating protein basigin/CD147/BSG (28), the multiple cell regulator CCN3/GOV (29), and the exosome marker molecule CD9 (30) also specifically altered between the variants and between the donors with no consistent expression pattern. LDHA levels in the cell supernatants served to control for necrotic cell death levels and were found to hardly vary (Fig. 3*B*).

##### Eicosanoid Formation in Metastatic Variants

Besides genes and proteins, lipids and especially eicosanoids with hormone-like activities were described to have the capability to affect and control metastatic processes (31). This is why the analysis of the cancer and metastasis promoting molecules PGE2 (32) and 12-HETE (33) was included in the present study. Indeed, significant alterations of the precursor molecule arachidonic acid as well as PGE2 and 12-HETE were observed between some C-CB pairs, again potentially contributing to a molecular classification system for metastatic properties. However, again none of these alterations showed uniformity regarding the metastatic variants and would thus be able to predict the given cell phenotype (Fig. 3*C*).

##### Redox Regulation in Metastatic Variants

**Fig. 4.**
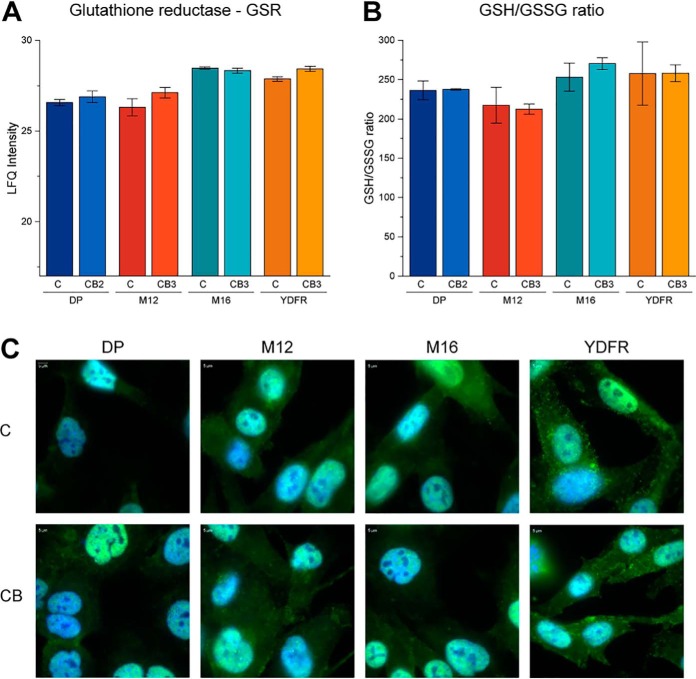
***A*, LFQ intensities of glutathione reductase (GSR) of cytoplasmic fraction.** *B*, Ratio of reduced (GSH) and oxidized (GSSG) form of glutathione (GSH/GSSG) of internal cellular intensities. *C*, Fluorescent microscopy shows the cellular distribution of NRF2 (green), co-stained with DAPI (blue) to indicate the nuclei.

Redox regulation is known to be highly relevant for metastasis and invasion (34). Thus, redox-regulating proteins as well as the ratio of reduced *versus* oxidized glutathione were investigated. Although no significant alterations were detected, the determined glutathione levels were found to remarkably parallel expression levels of glutathione reductase, an essential enzyme for glutathione metabolism (Fig. 4*A*, 4*B*). The transcription factor NRF2 is known to coordinate antioxidant responses in cells and mediates MAPK inhibitor resistance in melanoma cells (20, 35). Although not successfully identified by proteome profiling, we performed immunofluorescence staining and detected positive signals in all investigated melanoma cells (Fig. 4*C*). In accordance with the glutathione measurements, no characteristic difference in the localization of the transcription factor (nuclear/cytoplasmic ratio) was observable in any of the c-CB cell pairs.

##### Cell Morphology Assessment in Metastatic Variants

**Fig. 5.**
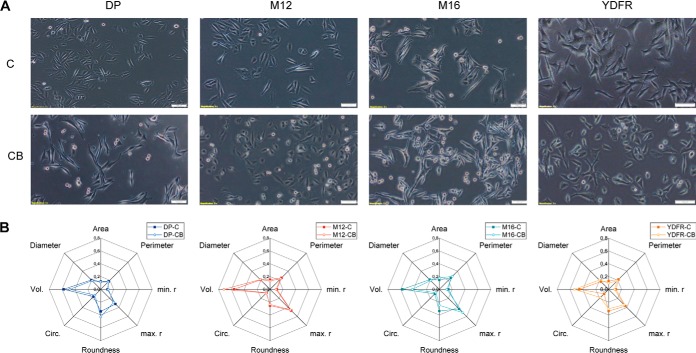
***A*, Morphological appearance of the melanoma variants; scale bars 100 μm.** *B*, Multiparametric morphological evaluation of melanoma cells: area [μm^2^], perimeter [μm], max. radius [μm], min. radius [μm], circularity, roundness, volume [fL] and diameter [μm].

On cell culture, some specific morphologic features of the cells were becoming apparent. EMT affects both cell morphology and the cells capability for metastasis (36). This motivated us to test the hypothesis that EMT-associated features might correlate with the different variants of metastasis. Eight different morphologic features of the cultured cells were determined manually. Microscopic images of the analyzed cells as well as spider webs depicting these selected morphologic characteristic are shown in Fig. 5. The spiderwebs revealed a similar tendency for all the 4 pairs (Fig. 5*B*) and in general a more “consistent” appearance for the cutaneous variants compared with the brain metastasis. However, although characteristic features were discerned, no uniform alterations were found to successfully distinguish cutaneous from brain metastases.

## DISCUSSION

The process of metastasis into distant organs such as the brain is complex, encompassing several steps including detachment of cells associated with ECM degradation, invasion through basement membranes into neighboring tissues, intravasation into the blood stream or lymphatic vessels and arrest and extravasation at a distal hospitable site to form the metastatic lesion (13). It is still not trivial to identify specific proteins or other molecules employed by the tumor cells to cope with these different kinds of challenges *in vivo* (37). Thus, it may be expected that it is hard to find a uniform molecular pattern associated with a given metastatic phenotype. The challenge is greatly aggravated by the known inter-tumor heterogeneity (38) as well as the plasticity of tumor cells in general (5) and of melanoma cells (16, 39). The challenge of inter-tumor heterogeneity underlying divergent clinical manifestations of different tumors belonging to the same histological type as well as distinct responses of such tumors to therapy is addressed by current precision medicine approaches (35, 40). The need for improved prediction capabilities out of molecular profiling data is thus evident.

The functional plasticity of tumor cells independent of genetic alterations is well recognized, rendering it conceivable that selection processes exerted by given *in vivo* conditions such as hypoxia, metabolic stress, biophysical stress or other tumor microenvironment (TME-) mediated effects may influence the properties of a developing tumor. We have previously aimed at the characterization of such kind of selection processes in case of chronic lymphocytic leukemia exerted via aging-related cell stress (15) and in case of multiple myeloma exerted via hypoxia (41).

The present model for brain metastasis, though associated with limitations as any other model, has major advantages to address these challenges. The cells derived from a common genetic ancestry were exposed to selection processes exerted by two different kinds of microenvironment via successive xenotransplantation homing into the sub-dermis or the cerebellum, respectively. The cells were subsequently grown *in vitro* at the same standard cell culture conditions and demonstrated to stably maintain different properties obviously related to their previous adaptation to the different kinds of microenvironment (Fig. 1*A*) (9). Thus the presently described differences between the local and metastatic (C and CB) variants represent intrinsic properties determined by their differential somatic genetic or epigenetic profile. These differences apparently remain independent of their actual microenvironment, as these two types of variants were maintained under standard cell culture conditions devoid of influences exerted by the original microenvironment. However, the original exposure to organ-specific microenvironments seems to have imprinted in the cells stable properties determining their metastatic phenotype.

Genetic traits, usually accounting for differences in stable phenotypes, do not always account for the potential to form metastasis in general and in the brain (42–44). If brain-metastasis drivers do indeed exist, we searched for molecular patterns that would differentiate local melanomas from corresponding brain metastasis. For this purpose we employed post-genomic techniques such as proteomics and eicosadomics as applied previously (22, 45).

Rather subtle alterations in the proteome profile were detected when comparing CB-C variants in case of two patients and more pronounced in case of the other two. However, indeed these alterations comprised many proteins known to be critical for mechanisms of metastasis.

This is the reason why we consider the present findings representative and relevant to the understanding of metastasis. Although a large variety of proteins including secreted proteins and lipid mediators, *i.e.* eicosanoids, were differentially expressed in local and brain metastatic variants, no characteristic pattern for metastatic properties emerged. If there was a common mechanism for brain metastasis, it seems that the presently employed analysis strategies were not yet able to discern it.

We suggest this observation is characteristic for a common but hardly recognized limitation of current molecular profiling analysis strategies. Functional cell properties based on rather linear molecule-to-function relations may be reliably classified, *i.e.* the expression of metalloproteinases directly mediates the capability of cell invasion. In contrast, complex cellular properties such as metastatic features potentially emerging from networks consisting of many interconnected molecules are hard to discern. Similarly, a photograph is composed of many pixels, associated with defined colors and signal intensity values. If the same pixels are stochastically scattered regarding their position, we lose the capability to interpret such a photograph correctly. We cannot interpret a photograph by the consecutive assessment of single data points, the relevant information can only be retrieved as meta-data emerging from the positional information of each pixel. In general, a molecular profiling data set contains quantitative information regarding a large number of distinct molecules. Although gene ontology classification allows us to relate most proteins to cellular properties, we are hardly capable of organizing the data points supporting improved retrieval of relevant meta-information as we can do in case of a photograph.

The present data strongly suggest there is no single regulatory event associated to a molecule which can determine metastatic properties of cancer cells. Future development of data interpretation strategies will demonstrate whether all the information contained in the present data would theoretically be enough to correctly classify the tumor cells.

### 

#### 

##### Conclusions and Outlook

To search for a molecular classification strategy discerning two different variants of metastasis we made use of a molecular profiling approach combined with morphological characterization of eight cell lines paired as cutaneous and brain metastasis. Focusing on the protein expression levels we were able to observe several significant proteomic profiles related to biological processes that are known to be essential for metastasis. Every cell line displayed specific molecular profiles that may contribute to the cell properties observed *in vivo*, but no classification strategy appeared yet suitable to classify the cells correctly. These observations are in line with the known plasticity and heterogeneity of tumor cells. We suggest that a common effort of the scientific community focusing on the development of improved data analysis strategies will be required to eventually qualify molecular profiling as a prediction tool supporting individualized precision medicine.

## DATA AVAILABILITY

The Proteomics data have been deposited to the ProteomeXchange Consortium (http://proteomecentral.proteomexchange.org) via the PRIDE repository and is available via the project accession PXD013765.

## Supplementary Material

Supplementary Figure S1

Supplementary Figure S2

Supplementary Figure S3

Supplementary Figure S4

Supplementary Table S1-S4

Supplementary Table S5

Supplementary Table S6

Supplementary Table S7

Supplementary Table S8

## Footnotes

^1^ The abbreviations used are:

FCS: fetal Bovine Serum
ACN: acetonitrile
CSC: cancer stem cells
CYT: cytoplasm
ECM: extracellular matrix
EMT: epithelial–mesenchymal transition
FA: formic acid
FDR: false discovery rate
GSH: glutathione (reduced)
GSSG: glutathione (oxidized)
12-HETE: 12-hydroxyeicosatetraenoic acid
NE: nuclear extract
NEM: N-ethylmaleimide
NP-40: nonidet P-40
PCA: principal component analysis
PGE2: prostaglandin E2
SIM: structured illumination microscopy
SN: supernatant
SPE: solid phase extraction
TCEP: tris(2-carboxyethyl)phosphine hydrochloride
TME: tumor microenvironment.
