# Supplementary material for: The Challenge of Classifying Metastatic Cell Properties by Molecular Profiling Exemplified with Cutaneous Melanoma Cells and Their Cerebral Metastasis from Patient Derived Mouse Xenografts
Source: Mol Cell Proteomics. 2019 Dec 31;19(3):478–89. doi: 10.1074/mcp.RA119.001886 (PMC7050108; doi:10.1074/mcp.RA119.001886)
Supplement: Supplementary Figure S1 [file 157378_0_supp_434650_q1jjw6.pdf]

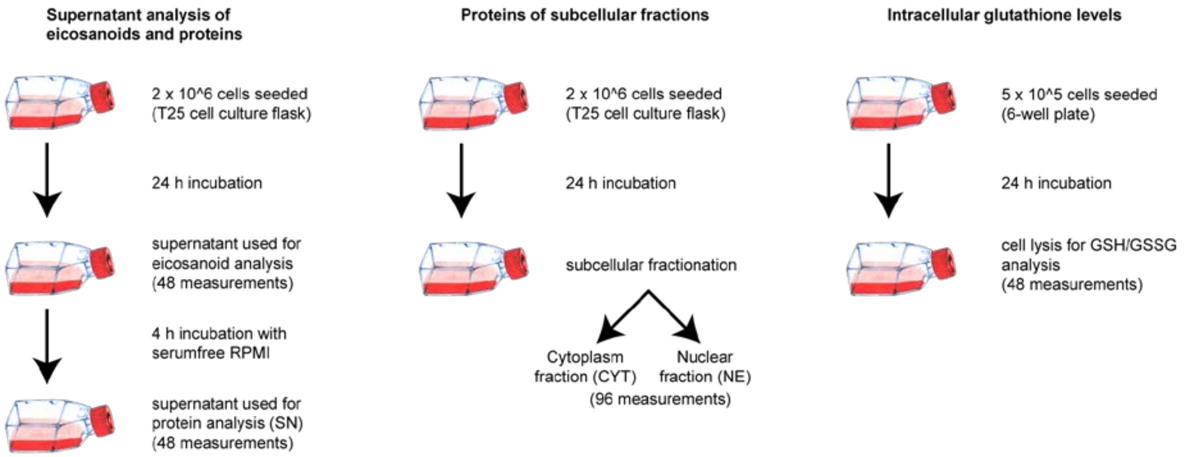

Supplementary Figure 1: Schematic experimental workflow for the molecular analysis of proteins, eicosanoids and glutathione.
