# Supplementary material for: The Challenge of Classifying Metastatic Cell Properties by Molecular Profiling Exemplified with Cutaneous Melanoma Cells and Their Cerebral Metastasis from Patient Derived Mouse Xenografts
Source: Mol Cell Proteomics. 2019 Dec 31;19(3):478–89. doi: 10.1074/mcp.RA119.001886 (PMC7050108; doi:10.1074/mcp.RA119.001886)
Supplement: Supplementary Figure S2 [file 157378_0_supp_434651_q1jjww.pdf]

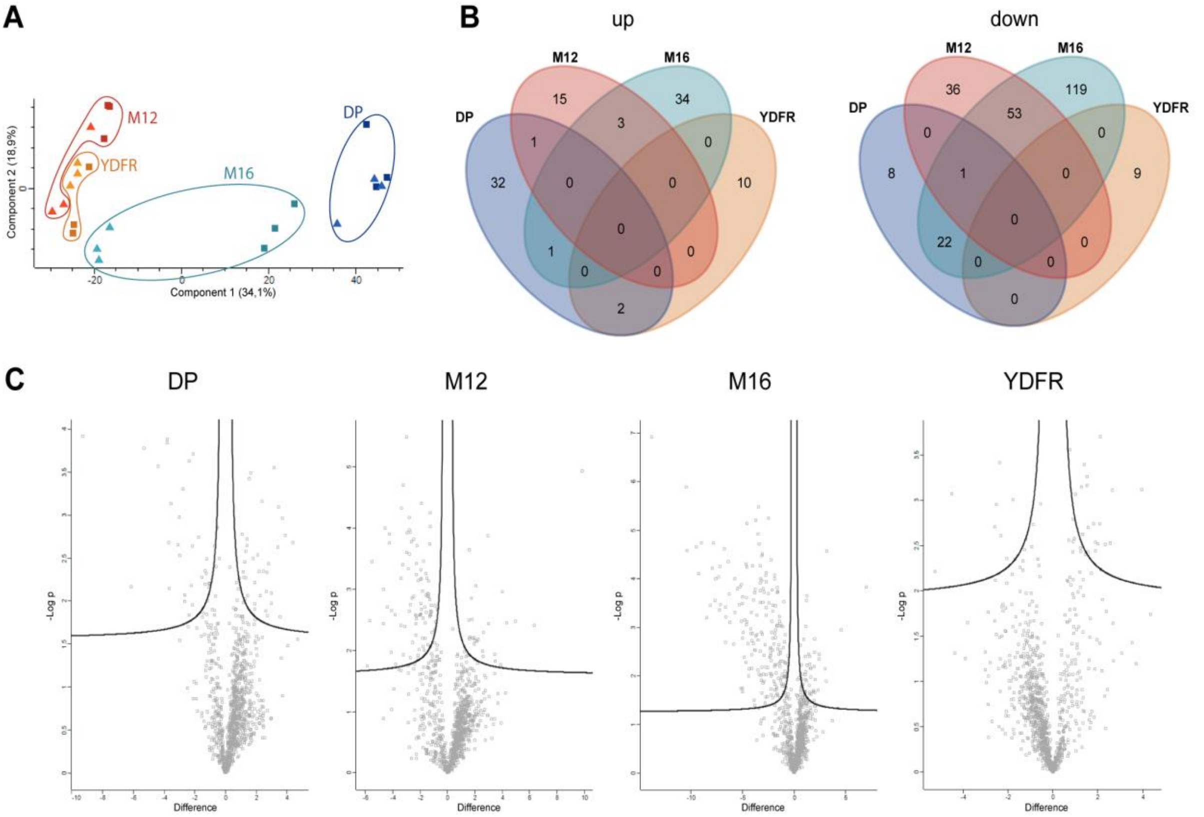

Supplementary Figure 2: (A) Principal component analysis (PCA) of the supernatant (SN). The C variants are represented by the rectangles while the CB variants are indicated by the triangles. **(B)** Venn diagrams comparing all significant regulations indicated in the volcano plots. The diagrams show the up- and down-regulated proteins from the supernatant separately. **(C)** Volcano plots for all 4 cell pairs comparing each C-CB pair (FDR = 0.05, S0 = 0.1). Up- or downregulation corresponds to higher or lower LFQ intensities in the CB variant compared to the C variant, respectively.
