# Supplementary material for: The Challenge of Classifying Metastatic Cell Properties by Molecular Profiling Exemplified with Cutaneous Melanoma Cells and Their Cerebral Metastasis from Patient Derived Mouse Xenografts
Source: Mol Cell Proteomics. 2019 Dec 31;19(3):478–89. doi: 10.1074/mcp.RA119.001886 (PMC7050108; doi:10.1074/mcp.RA119.001886)
Supplement: Supplementary Figure S3 [file 157378_0_supp_434652_q1jjw6.pdf]

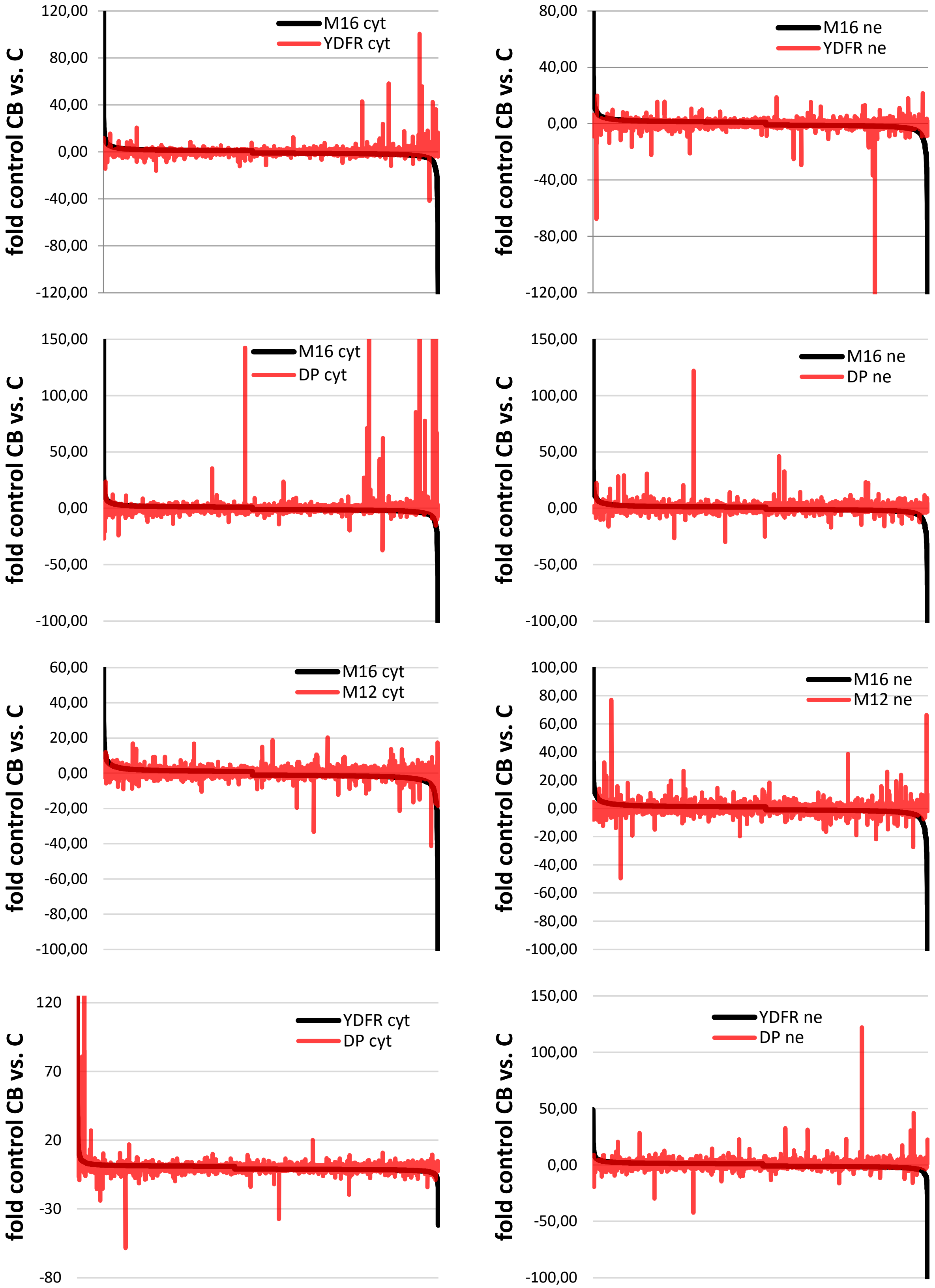
fold control CB vs. C
120,00
80,00
40,00
0,00
-40,00
-80,00
-120,00
M16 cyt
YDFR cyt
fold control CB vs. C
80,00
40,00
0,00
-40,00
-80,00
-120,00
M16 ne
YDFR ne
fold control CB vs. C
150,00
100,00
50,00
0,00
-50,00
-100,00
M16 cyt
DP cyt
fold control CB vs. C
150,00
100,00
50,00
0,00
-50,00
-100,00
M16 ne
DP ne
fold control CB vs. C
60,00
40,00
20,00
0,00
-20,00
-40,00
-60,00
-80,00
-100,00
M16 cyt
M12 cyt
fold control CB vs. C
100,00
80,00
60,00
40,00
20,00
0,00
-20,00
-40,00
-60,00
-80,00
-100,00
M16 ne
M12 ne
fold control CB vs. C
120
70
20
-30
-80
YDFR cyt
DP cyt
fold control CB vs. C
150,00
100,00
50,00
0,00
-50,00
-100,00
YDFR ne
DP ne

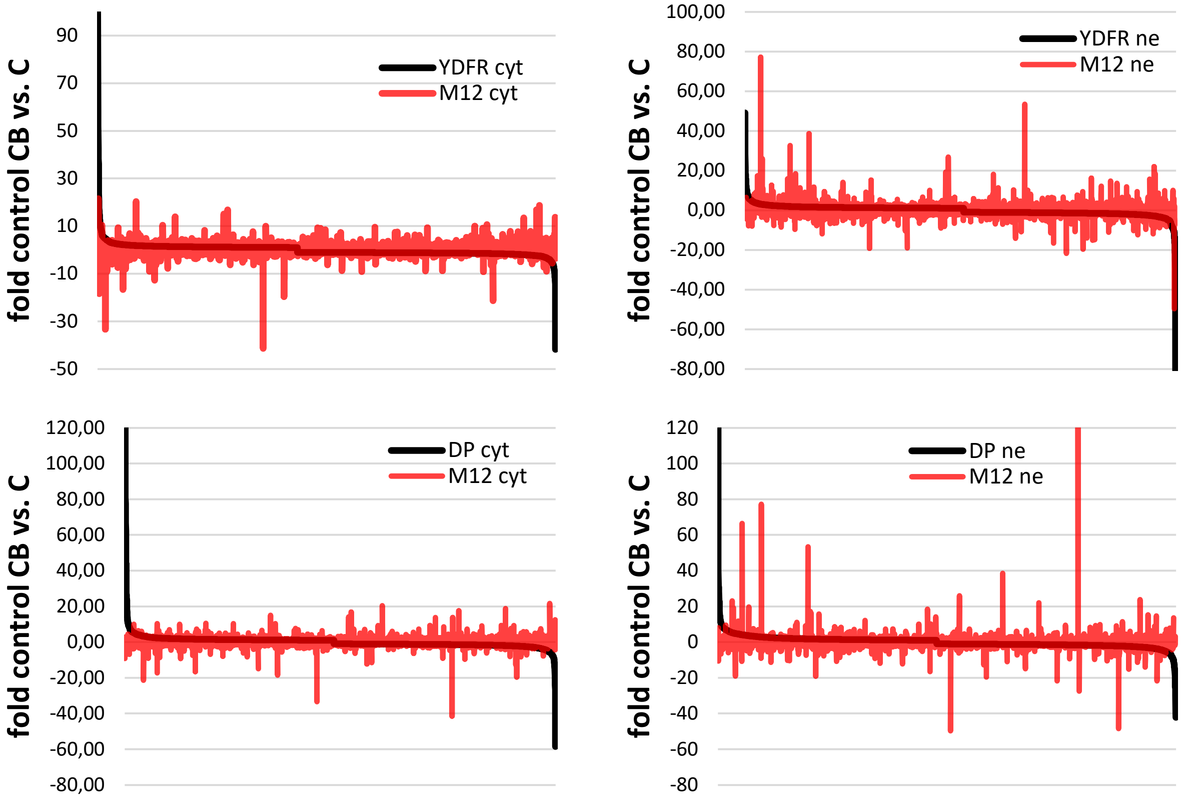

Supplementary Figure 3: Differences in protein regulation of cell pairs. Fold-change values between cerebellar (CB) and cutaneous (C) variants of a cell pair were sorted in a descending fashion, separately for cytoplasmic (cyt) and nuclear (ne) proteins. The corresponding fold-change values of another cell pair were blotted in red. A positive correlation of deregulated proteins would be visible by alignments between black and red lines especially at the edges. Figures were created using Excel 2010.
