# Supplementary material for: The Challenge of Classifying Metastatic Cell Properties by Molecular Profiling Exemplified with Cutaneous Melanoma Cells and Their Cerebral Metastasis from Patient Derived Mouse Xenografts
Source: Mol Cell Proteomics. 2019 Dec 31;19(3):478–89. doi: 10.1074/mcp.RA119.001886 (PMC7050108; doi:10.1074/mcp.RA119.001886)
Supplement: Supplementary Figure S4 [file 157378_0_supp_434653_q1jjwd.pdf]

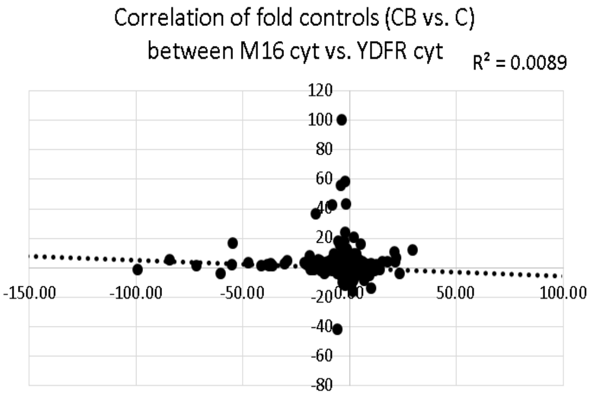
Correlation of fold controls (CB vs. C)
between M16 cyt vs. YDFR cyt
R² = 0.0089
120
100
80
60
40
20
-20
-40
-60
-80
-150.00
-100.00
-50.00
50.00
100.00

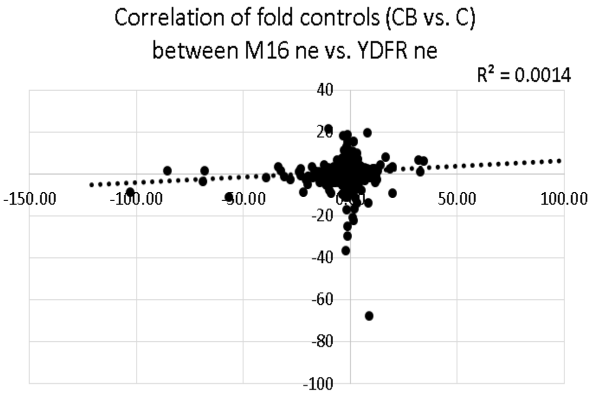
Correlation of fold controls (CB vs. C)
between M16 ne vs. YDFR ne
R² = 0.0014
40
20
-20
-40
-60
-80
-100
-150.00
-100.00
-50.00
50.00
100.00

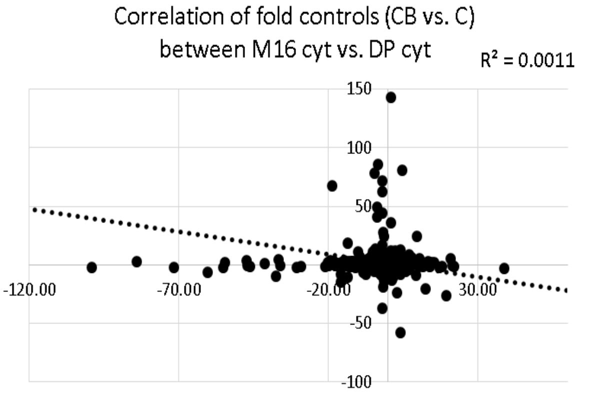
Correlation of fold controls (CB vs. C)
between M16 cyt vs. DP cyt
R² = 0.0011
150
100
50
-50
-100
-120.00
-70.00
-20.00
30.00

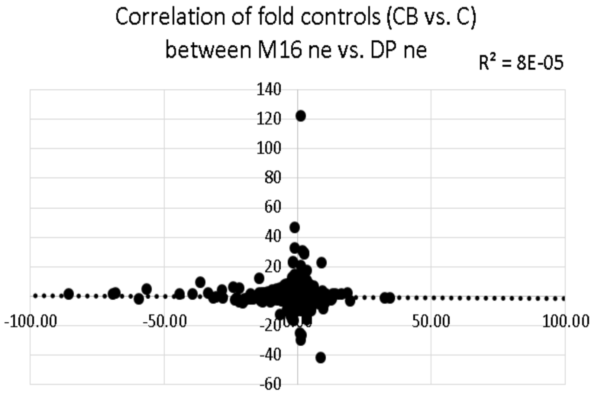
Correlation of fold controls (CB vs. C)
between M16 ne vs. DP ne
R² = 8E-05
140
120
100
80
60
40
20
-20
-40
-60
-100.00
-50.00
50.00
100.00

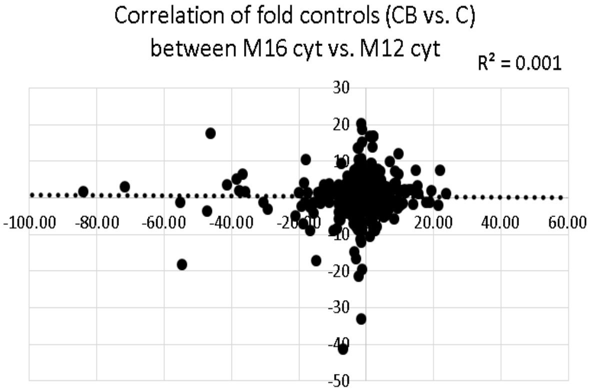
Correlation of fold controls (CB vs. C)
between M16 cyt vs. M12 cyt
R² = 0.001
30
20
10
-10
-20
-30
-40
-50
-100.00
-80.00
-60.00
-40.00
-20.00
20.00
40.00
60.00

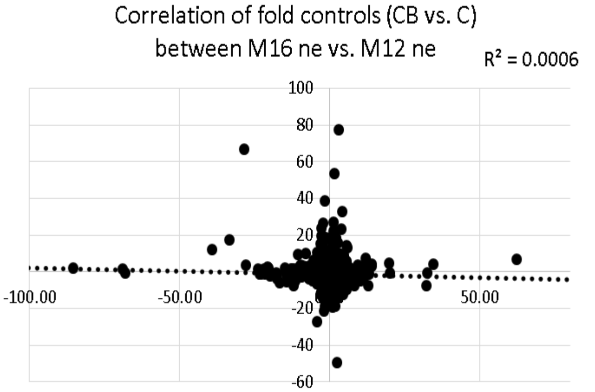
Correlation of fold controls (CB vs. C)
between M16 ne vs. M12 ne
R² = 0.0006
100
80
60
40
20
-20
-40
-60
-100.00
-50.00
50.00

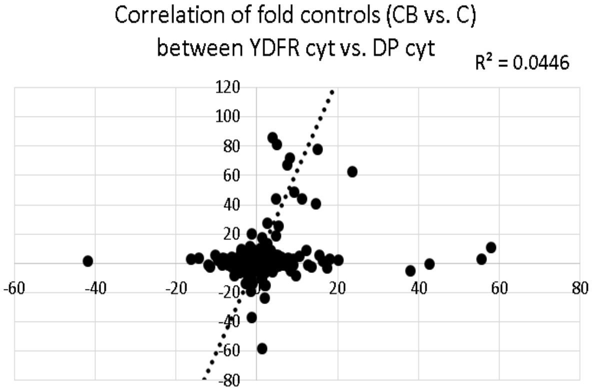
Correlation of fold controls (CB vs. C)
between YDFR cyt vs. DP cyt
R² = 0.0446
120
100
80
60
40
20
-20
-40
-60
-80
-60
-40
-20
20
40
60
80

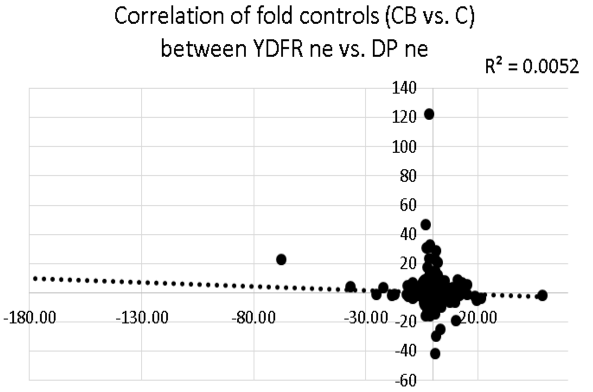
Correlation of fold controls (CB vs. C)
between YDFR ne vs. DP ne
R² = 0.0052
140
120
100
80
60
40
20
-20
-40
-60
-180.00
-130.00
-80.00
-30.00
20.00

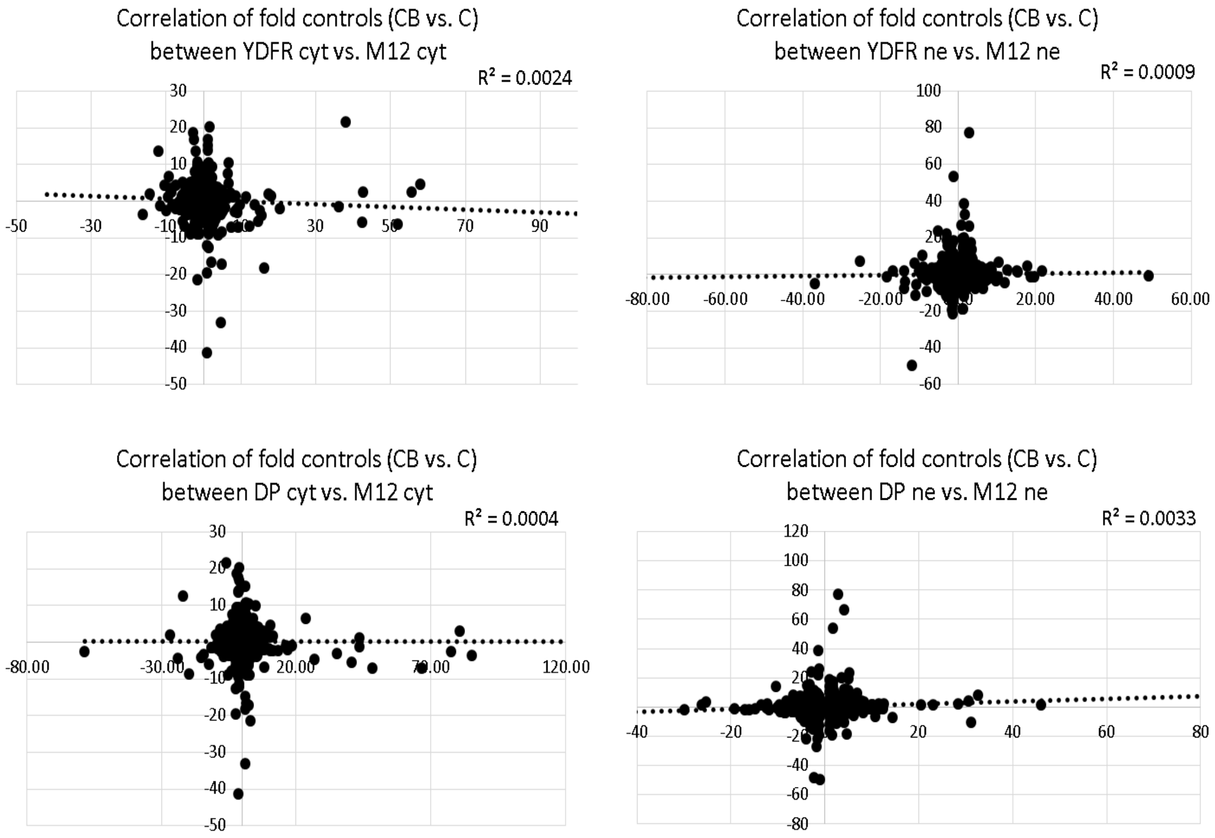

Supplementary Figure 4: Correlation analysis of differential expression values of proteins. Correlation analysis of fold-change values between cerebellar (CB) and cutaneous (C) variants was performed for cell pairs as indicated, separately for cytoplasmic (cyt) and nuclear (ne) proteins. Graphs were created using Excel 2010.
