# Supplementary material for: The Challenge of Classifying Metastatic Cell Properties by Molecular Profiling Exemplified with Cutaneous Melanoma Cells and Their Cerebral Metastasis from Patient Derived Mouse Xenografts
Source: Mol Cell Proteomics. 2019 Dec 31;19(3):478–89. doi: 10.1074/mcp.RA119.001886 (PMC7050108; doi:10.1074/mcp.RA119.001886)
Supplement: Supplementary Table S1-S4 [file 157378_0_supp_434654_q1jjwj.pdf]

Supplementary Table S1: Eicosanoid Standards; All eicosanoid standards were bought from Cayman Europe, Tallinn, Estonia.

Standard Mixtrue	Name	Abbreviation	Conc. Stock solution [nM]	Final conc. [nM]
Standards spiked **before** solid phase extraction	12S-Hydroxyeicosatetraenoic acid d8	12S-HETE-d8	609,2	20,3
	15S-Hydroxyeicosatetraenoic acid d8	15S-HETE-d8	609,2	20,3
	5-Oxo-Eicosatetraenoic acid d7	5-Oxo-ETE-d7	1844,6	61,5
	11,12-Dihydroxy-5,8,14-icosatrienoic acid d11	11.12-DiHETrE-d11	1122,8	37,4
	Prostaglandin E2-d4	PGE2-d4	613,0	20,4
	20-Hydroxyeicosatetraenoic acid d6	20-HETE-d6	572,5	19,1
Standards spiked **after** solid phase extraction	5S-Hydroxyeicosatetraenoic acid d8	5S-HETE-d8	609,2	20,3
	14,15-Dihydroxy-5,8,11-icosatrienoic acid d11	14.15.DiHETrE-d11	572,5	19,1
	8-Isoprostaglandin F2α d4	8-iso-PGF2α-d4	1116,5	37,2

Supplementary Table S2: Inclusion list of eicosanoids as used for the MS/MS analysis

Mass [m/z]
254,2245
275,2011
277,2167
279,2324
281,2480
283,2637
293,2122
295,2279
301,2168
303,2330
311,2228
315,1966
317,2122
319,2279
321,2435
327,2324
327,2781
329,2480
333,2071
335,2222
337,2384
343,2279
348,3069
349,202
351,2177
353,2328

355,2428	
357,2585	
359,2222	
367,3576	
375,2171	

Supplementary Table S3: m/z values of MRM transitions with collision energies (CE) for GSH, GSSG, GSH-NEM, GSH-d5NEM, NEM and d5NEM

Analyte	Q1	Q3	CE
GSSG	613.2	484.1	20
	613.2	595.1	25
	613.2	355.1	30
GSH	308.1	233.2	15
	308.1	179	19
	308.1	76.2	47
GSH-NEM	433	201	35
	433	**84.2**	59
GSH-d5NEM	438	206	35
	438	**84.2**	59
NEM	126	98	19
	126	80	25
d5NEM	131	98	19
	131	80	25

Supplementary Table S4: Parameters for the morphological evaluation of the melanoma cells variants.

Parameter	Data	unit	Adjustment factor
Area	cellSens Entry	[µm²]	1/10000
Perimeter	cellSens Entry	[µm]	1/1000
Radius (min)	cellSens Entry	[µm]	1/100
Radius (max)	cellSens Entry	[µm]	1/100
Circularity	π *Area/ Perimeter2	-	-
Roundness	r(min)/r(max)	-	-
Volume	CASY-TT	[fL]	1/10
Diameter	CASY-TT	[µm]	1/100
